# Implementation evaluation of a leadership development intervention for improved family experience in a private paediatric care hospital, Pakistan

**DOI:** 10.1186/s12913-022-08342-2

**Published:** 2022-07-23

**Authors:** Muneera A. Rasheed, Ayesha Hussain, Amin Hashwani, Johannes T. Kedzierski, Babar S. Hasan

**Affiliations:** 1 Department of Paediatrics & Child Health, Aga Khan University, Karachi, Pakistan; 2 Charter for Compassion, Karachi, Pakistan; 3 Aga Khan University Hospital, Karachi, Pakistan

**Keywords:** Leadership development, Patient experience, Employee engagement, Implementation evaluation, Social media data

## Abstract

**Background:**

A study from a tertiary care center in Pakistan demonstrated that a leadership development intervention led to improved family experience of care outcomes. The objective of the current paper is to assess the implementation of this intervention and identify barriers and facilitators to inform sustainability and scalability.

**Methods:**

A working group designed the intervention using a theory-of-change model to strengthen leadership development to achieve greater employee engagement. The interventions included: i) purpose and vision through purpose-driven leadership skills trainings; ii) engaging managers via on-the-job mentorship programme for managers, iii) employee voice i.e., facilitation of upward communication to hear the employees using Facebook group and subsequently inviting them to lead quality improvement (QI) projects; and iv) demonstrating integrity by streamlining actions taken based on routine patient experience data. Implementation outcomes included acceptability, adoption, fidelity across degree & quality of execution and facilitators & barriers to the implementation. Data analyzed included project documentation records and posts on the Facebook group.

Analysis indicated acceptability and adoption of the intervention by the employees as178 applications for different QI projects were received. Leadership sessions were delivered to 455 (75%) of the employees and social media communication was effective to engage employees. However, mentorship package was not rolled out nor the streamlined processes for action on patient experience data achieved the desired fidelity. Only 6 QI projects were sustained for at least a year out of the 18 approved by the working group. Facilitators included leadership involvement, real-time recognition and feedback and value-creation through participation by national and international celebrities. Challenges identified were the short length of the intervention and incentives not being institutionalized.

The authors conclude that leadership development through short training sessions and on-going communications facilitated by social media were the key processes that helped achieve the outcomes. However, a long-term strategy is needed for individual managerial behaviours to sustain.

**Supplementary Information:**

The online version contains supplementary material available at 10.1186/s12913-022-08342-2.

## Introduction

Patient-centered care measured by patient and family experience is one of the core values of high-quality health systems [1]. Improving patient and family experience is integral to ensuring satisfaction and continued use of the services, a priority for many private service providers [2–4]. As per the findings of a survey done by the Beryl Institute of Patient Experience across US hospital, employee engagement was rated as the most important factor in achieving positive patient experience [5]. This highlights the fact that health care organizations have begun to recognize the role of their staff in driving the patient experience [6]. Hence, interventions to enhance patient experience require more than an emphasis on appropriate policies and collecting patient feedback [7]. It is vital to create a culture that values provision of optimal human experience through intrinsic and extrinsic reward system leading to engaged employees [8]. One of the critical enabling factors for shaping the culture of health systems is leadership [9]. Leadership has also been recognized as a one of the major factors for effective governance of health systems by the World Health Organization [10] and Alliance for Health Policy and Systems Research [11].

The leveraging role of leadership skills is of particular importance in low and middle-income countries (LMIC) when functioning with constrained resources. Burden of disease, dearth of high skilled care providers, limited access to adequately equipped health care facilities in such settings creates a culture where patient experience does not remain a high priority [12]. However, an urgent need to improve the quality of health care systems in these settings is now being recognized with user experience as one of the key indicators [13]. This also alludes to the need to change the doctor-centric culture (characterized by a culture where doctors sit at the top of the hierarchy and tend to be the main decision-makers) to a patient-centric one where patient reported outcomes and experience will become a significant health care system performance metric [14]. This change in culture will also create challenges for health care leaders in LMICs who either are medical officers in charge of health units, often promoted on account of their clinical expertise alone and rarely have any prior leadership training or administrators trained on the lines of a physician centric culture [15]. Coupled with overall weak governance in the system, they tend to follow the redundant leadership strategies. Hence, leadership of healthcare organizations in LMIC is often characterized as authoritarian and hierarchical, reducing its effectiveness [16]. It is also realized that such practices affect staff motivation subsequently leading to poor patient care and experience [17]. Improving management and leadership practices specifically leading to a culture of improved patient and employee experience means challenging and upsetting the traditional hierarchies [18], thus while designing any change intervention, complex workplace dynamics should be kept in mind [19]. An approach that would require not building individual leaders but an effort to improve the organizational leadership-“*the collective capacity of the organization to engage people in leadership roles”* [20]. This cannot happen through leader development but *leadership development*. While the former is focused on strengthening personal and emotional traits of individual to enable them to perform better in their formal roles, the latter takes into account the subtle role of informal influence and building strong relationships to enable dialogue and difficult conversations for change [8, 9, 19]. Leadership development through inclusion of long-term mentorship and external support has been recommended (details under supplementary file 1) [21]. Enhancing patient experience, therefore, would mean the leadership co-constructing shared values from the lens of human vulnerability in order to engage workforce for the common goal [22, 23].

Utilizing the action learning approach for leadership development similar to the work by Accoe et al. (2020) built on empowering frontline staff and capacity-building [24], an intervention study (the Patient experience Initiative-PExpI) was conducted at the Aga Khan University Hospital (AKUH) [25]. The findings indicated benefits by demonstrating improved family experience. Additionally, it showed that families exposed to the intervention were three times more likely to recommend the hospital [25]. Being a unique, leadership-focused study in a LMIC healthcare context that showed benefits despite resource constraints, it is important to unpack the implementation of PExpI to inform sustainability, scalability and replication in other similar contexts. Hence, the objective of this current paper is to assess the implementation of the PExpI intervention to determine if PExpI was implemented as intended and to identify what components worked or did not work and why.

## Methods

### Setting

The AKUH is a Joint Commission International (JCI)-accredited private hospital located in Karachi, the largest city of Pakistan. The institution serves two provinces i.e., Sindh and Balochistan (populations of 47.89 million and 12.34 million, respectively) for complex care. The PExpI was carried out in the paediatrics service line (SL) at AKUH. The inpatient, catering to children from birth to 18 years consists of a paediatric ward with two acute care units comprising 122 beds, a neonatal unit with facility of admitting 24 neonates along with intensive care unit offering support to 8 children at a time and a round-the-clock emergency care. Every month, an estimated 500 patients are seen in the acute care ward. The services are overseen by a Service Line Chief (SLC) who is supported by a Business Manager and a Nursing Manager. The SLC of the different service lines report to the CEO. The pediatric service line includes about 600 employees, ~ 60% belonged to nursing services, 11% were physician faculty members, 13% from administration, and 12% were trainee physicians.

### Study design

The current study was designed as a mixed method evaluation of the PExpI intervention. It was conducted between October 2017 to December 2020 with the intent to provide a more comprehensive account than either a quantitative or qualitative assessment alone. Following the concurrent mixed method approach, each method was conducted and analyzed separately, and findings were later triangulated [26]. We used the implementation evaluation framework by Proctor et al. (2011) [27]. The domains of interest have been summarized in Table 3. Through this evaluation we examined the following implementation outcomes of the PExpI intervention:

- acceptability defined as the evaluation of the employees’ reaction to the intervention and the extent to which they consider the strategy to be appropriate and satisfactory; and
- adoption defined as the extent to which the intervention strategy was likely to be used by the employees
- fidelity i.e., degree and quality of execution defined as the extent, probability and manner in which the intervention is executed as planned,
- barriers and facilitators to acceptability, adoption or implementation.

The study was approved as a QI project by the institutional Ethics Review Committee of the Aga Khan University.

### The PExpI intervention

#### Development approach

The intervention was designed using a combination of approaches informed by guiding principle set by the team [28]:

- target population: people who will use the intervention i.e., the employees. Interventions were designed on the views and perspective of the target population.
- theory and evidence based: interventions will be informed by published research evidence and existing theories e.g., organizational development theories, and
- implementation: designing with attention to execution in the setting e.g., aligning roles with job description as much possible to enhance operational feasibility at the outset.

The development and design of the intervention are described below across different steps.

#### Conceptualization and creation of a working group

The new leadership of the paediatric service line in April 2017 realized that the current quality framework consisted only of provision of care and system efficiency (as required by JCI indicators) but did not include a definition of quality from a patient’s perspective. The responsibility to assess this ‘patient perception’ of quality and status of their experience was assigned to a behavioral implementation scientist (MR) in the SL. The assessment began with a qualitative analysis of patient and family feedback forms available as part of the institutional procedures. These findings indicated that the families felt the staff was disengaged and was unable to responds to their emotional and informational needs. Further details are available in the supplementary file 1, Table S1. These observations were shared with the hospital leadership who recognized the need of an employee engagement intervention. The intervention study was henceforth conceptualized by the SLC and the behaviour scientist (MR). A working group was subsequently created to have a mix of people who were in leadership and operational roles and also domain experts to steer the initiative. The final group included the SLC, the behaviour and implementation scientist (MR), service academic leader, business and nursing managers, a leadership coach, and a communication consultant.

#### Identification of a conceptual framework and assessment of preconditions

Following the hypothesis that the main driver of patient experience is employee engagement, the current study adopted a framework from organizational theory (also see supplementary file 1). MacLeod & Clarke (2009) [29] framework identifies four enablers: leadership (setting purpose and vision), engaging managers (ensuring engagement of middle managers), employee voice (hearing employees and providing them autonomy to resolve their challenges), and integrity (demonstrating integrity through transparent and fair recognition of high-performing values).

The assessment of the preconditions was completed through reflections of the working group with the leadership coach and employee pain point surveys (based on one item which required the employee to list down the three top pain points of their experience). The group identified a lack of organizational competition in the market as a barrier to initiating interventions for enhancing employee engagement. In terms of incorporating patient experience, there was a designated department that works on handling patient complaints, which, by definition was a reactive approach to resolve patient complaints, leading to failure in addressing problems and hence, blurring the need for engaging employees. An analysis of pain point survey of 256 employees: physicians (*N* = 22), trainee physicians *(N* = 77), and nursing staff (*N* = 157) revealed three overarching themes: lack of connection with work, feeling overburdened, and lack of growth opportunities (Table 1).

**Table 1:** Assessment of preconditions for employee engagement based on framework by MacLeod & Clarke (2009) [29]

Needs	Assessment
Purpose and vision	The working group felt the new hospital leadership that took charge in 2015 had a clear vision and a strategic plan for improving patient care. This was perceived to be a critical factor to enable implementation of employee engagement strategy. The same strategic vision now required communication and translation downstream within the level of each service line to ultimately benefit the patient
Engaging managers	Senior physicians expressed feeling stretched to fulfil both academic and service obligations thus highlighting the need of on-going mentorship. Analysis of the pain points reported by faculty physicians (*N* = 22) revealed that the most reported theme was a perception of unfairness based on their workload, compensation, timely promotions (60%), followed by lack of vision (25%) and growth opportunities (20%). About 15% of staff signified lack of respect and teamwork to be another pain point The data from nursing (*N* = 157) indicated that the main challenge was related to work related issues (57%) comprising work load, lack of facilities, salary, staff: patient ratio, double duties and excessive documentation indicating the need for a greater efficient management. The next theme was related to their emotional experience (17%) including lack of appreciation, favoritism, lack of structured routine and breaktime. Discussion with the staff revealed that no structured supervision strategy for staff or administrative expectations were established. Staff’s growth was not included in the supervisors’ appraisal nor was any training as supervisors provided as part of their job. For trainee physicians (*N* = 77), dissatisfaction with academics was one of the key pain points (62.5%) followed by not being granted enough leaves (36%). Issues such as lack of trust (25%) compassion (25%), and appreciation (20%) were also indicated
Employee voice	There are HR (Human Resource) policies for grievance and harassment but there is none for ongoing feedback. Employee voices can remain unheard because there was no system to share routine feedback and access to senior management was also a challenge. However, with the newly appointed hospital leadership there was a renewed interest in engaging staff for their resolutions but no strategy was yet designed
Integrity (alignment between policy and value system)	The working group concluded that while the hospital policy valued patient care and experience, the culture did not specifically value the employee providing greater patient experience. They also felt that it was important for the employees to be appraised on provision of service excellence. This was evident from the fact that patient appreciations received were not communicated to the employee nor was their due recognition of such practices

#### Intervention design and procedures

The working group formed a theory of change (ToC) [30] model for reorganizing the framework of patient experience of care [supplementary text]. The overall purpose of the framework was to enhance patient-centered practices by introducing interventions to improve employee engagement and addressing their pain points. The working group concluded that a holistic framework was needed that would cover the four enablers i.e. purpose and vision, engaging middle managers, giving employees a voice and demonstrating integrity, for meaningful and sustainable engagement. The eventual goal was to improve the overall patient experience as indexed by willingness to recommend the hospital [25] through implementation of different QI projects informed by patient and employee pain points [31]. A set of respective interventions were identified emerging from the conceptual framework and operationalized using ToC to achieve the eventual goal of improving patient and family experience [Table 2]. The intervention procedures included short leadership workshops based on approach by Tony Robbins, a US based business consultant [32]. The leadership coach recruited for the study was based in Pakistan but had attended trainings by Tony Robbins. Being familiar with the context he was able to customize the trainings according to the needs of the participants e.g., he included examples of local leaders in Pakistan (building on his previous work). Rest of the procedures included a combination of group discussions and regular meetings of the working group; development and subsequent roll out of a compassionate mentorship package for employees in a managerial role [33], social media-based communication platform [34] and standard procedures for recognition of high performing employees [35]. The overarching approach to PExpI was emulation of a purpose driven agenda through shared communication and empathizing with each other and scaling the individual transformation sessions to an entire paediatric service unit facilitated by technology utilizing implementation science strategies e.g., facilitation, creating a formal blueprint and creating demand based on guidelines by Powell et al. 2015 [36]. The sessions were live streamed allowing employees to view real time in their space along with an opportunity to record their feedback as comments or reactions. Similar conversations also continued on the social media led by working group and leadership coach as different posts. Refer to supplementary file 1 (Table S2) for details.

**Table 2:** Preconditions and interventions identified for employee engagement for the initiative

Enabler	Description	Preconditions for patient experience	Interventions	Metrics for degree & quality of execution
Purpose and vision	Visible leadership ensures a strong, transparent and explicit organizational culture by providing a strategic narrative while maintaining transparency. Narrative is communicated in a way to make employees feel part of it	1) leadership is visible 2) leadership communicates a strategic vision of patient experience; 3) leaders value patient experience and consistently communicate so	1) ensure leadership is present and visible in the initiative activities; 2) integrate the patient experience indicators into the quality framework and train senior managers to communicate the strategic narrative around the vision of patient experience; 3) create a platform to communicate the narrative with employees and answer their questions at formal and informal level	Degree: -Number of posts and comments by leadership on Facebook group -Number of posts around patient-centricity -Attendance of leadership (any working group member) at training sessions of the managers and employees (whether in group or individual training session). Leadership here will be classified as either CEO, CMO, SLC, Chair, BM, NM or director patient experience Quality -Thoughtfulness and engagement generated by the posts
Engage managers	Managers are engaged as they are vital to engaging employees but also inspiring, motivating and coaching them. They are considered to be at the heart of organizational culture	4) leadership can engage (line) managers for the new vision and clearly communicate about their roles and expectations; 5) managers can efficiently design workload of their employees; 6) managers treat their employees with respect; 7) managers are able to provide constructive feedback to intellectually stretch employees; 8) managers are able to create a mentor–mentee relation rather than a supervisor-supervisee one	4) provide training to line managers to engage them with the new vision; 5–7) develop and implement a compassion-based mentorship package for the supervisors/line managers enabling them to provide supportive supervision to their frontline staff for respectful and empathetic communication skills;	Degree -Creation of mentorship module -Leads from SL engaged the higher leadership -Number of man-hours spent in creating the mentorship module -Pilot already done with feedback from the pilot used to modify the module Quality -The depth of created module -Creating dashboards and adopting technology to facilitate its implementation -Promotion structure developed around principles of mentorship to value this intervention
Employee voice	Employees are given autonomy to voice their concerns and needs and to be able to express how they do their job and in decision-making in their own department along with sharing any occurring problems and challenges with commitment to arrive at joint solutions	9) employees have access to and can share work-related challenges with the managers and leadership 10) employees have the autonomy to co-design solutions for the problems	8) create an on-line, real-time platform to facilitate upward communication facilitating employees to share their challenges; 9–10) encourage employees to be part of designing and implementing solutions through initiation of QI projects	Degree -Number of intervention plans made, number of plans initiated, number of plans sustained Quality -Optimal quality intervention = intervention which had clear ToC, process metrics and outcome metrics defined and were on track (need to meet all criteria to be called optimal) -Adequate quality intervention = clear ToC, at least 50% process and outcome metrics defined and being collected and 50% on track (need to meet at least 2 criteria) -Inadequate quality intervention = no ToC, < 50% outcome or process metric defined, < 50% on track (any one of the criteria will make one inadequate)
(Organizational) integrity	Organizational values are reflected in day-to-day behaviours. Creating a belief in employees that the organization is true to its values, maintains equity, sets and reinforces behaviour expectations	11) patient experience is objectively measured and metrics are regularly communicated to employees; 12) patient experience is part of employee performance review system and promotion criteria; 13) managers regularly recognize high-performing employees based on a fair recognition system;	11) streamline patient experience data & metrics, creating SOPs to routinely measure and monitor patient experience data; 12) give due weightage to patient experience-related performance in the appraisal 13) set a performance-based criteria to recognize staff who received appreciation from patients for providing adequate experience to patients on the service line	Degree -The SOPs created and if patient experience incorporated in appraisal -Person of the week and meeting celebrity was tied to appraisal which involved patient feedback Quality -Details of SOP (Please refer to supplementary material-file 2) -Time to appreciate employee by the respective managers after an appreciation was received

*Note: CEO* Chief Executive Officer, *BM* Business Manager, *CMO* Chief Medical Officer, *NM* Nursing Manager, *SLC* Service Line Chief, *SOP* Standard Operating Protocols, *ToC* Theory of Chan

### Data analysis

The implementation outcomes and sources of data have been summarized in Table 3. Part of a service coordinator’s time was dedicated to the PExpI for record-keeping of the implementation data including attendance and project updates. The data on Facebook group was managed by a communication consultant initially and later the responsibility was handed to a research associate (organizational psychologist). A total of 9085 posts with over 60,000 comments from the Facebook group were analyzed. Due to change in Facebook group privacy rules in 2018, it was not possible to disaggregate data by individual employees. Data from the first 6 months (October 2017 to March 2018) showed that of the total members 50% reacted, 25% commented and 10% posted. However, posts were viewed by more than 90% of the members (which remained the same throughout the PExpI). Qualitatively, in the early phase of the intervention the majority of the data was from the faculty physicians and senior managers. Overtime the frontline staff also started sharing their feedback and mostly on the appreciation posts (e.g., person of the week). The quantitative indicators included the number of active members, total posts uploaded with mean comments and reactions per post. Qualitative analysis followed a deductive approach [37]. The coding framework was informed by the MacLeod & Clark (2009) [29] model of employee engagement and Proctor et al. (2011) implementation concepts [27]. The qualitative analysis team included three members. One was the lead investigator to provide intervention implementation insights and the other two research assistants (RA), graduates in psychology provided a perspective from organizational psychology and compassionate behaviours in healthcare. This also served to minimize bias as these RA joined after the active intervention period and one was employee of the partner organization (Charter for Compassion, Pakistan), thus bringing a perspective from the lens of a third-party analyst of the intervention. The posts were first coded by two members independently. These members did not include the lead investigator as it would have created bias as the lead investigator was in the project from the very beginning and was also aware of the roles and responsibilities of the participants. Though the RA knew identity of the participants but were not aware of the specific roles and responsibilities or the context in which these participants operated within the organization. The coding was agreed in a separate meeting to ensure validity. Narratives were created by the RA and were finalized by the lead investigator (MR). The quotes presented in the findings were selected to represent the narrative [38]. Qualitative findings related to different aspects of implementation (e.g., acceptability) were triangulated with quantitative indicators e.g., percentage by cadres of attendance and participation in initiative activities including workshops, number of QI projects shortlisted and implemented, and ratings of the progress of QI projects.

**Table 3:** Research question and outcome of interest for each domain

Areas of Focus	Questions	Outcome of interest	Source of Data
**Acceptability** evaluates the participants’ reaction to the intervention and the extent to which they consider the strategy to be appropriate and satisfactory	To what extent was the intervention perceived as suitable, satisfying, or attractive to the employees and the implementers?	Satisfaction with experience and content	Employee feedback (Facebook group data)
**Adoption** aims to answer the extent to which the intervention strategy was likely to be used	To what extent was intervention used (i.e., how much demand was likely to exist?)	Expressed intent of use	Employee feedback (Facebook group data)
**Fidelity** determines the extent, probability and manner in which the intervention is executed as planned	To what degree was the intervention executed as planned and executed with quality?	Degree and quality of execution of interventions for each component	Attendance records and leadership coach notes Records of QI projects implemented and sustained Qualitative data (Facebook group data)
**Barriers and facilitators** aim to explore the barriers and facilitators to execution, acceptability or adoption	What were the barriers and facilitators to implementation?	Barriers and facilitators Amount, type of resources needed to implement	Team and consultant notes Qualitative data (Facebook group data)

*Note: QI* Quality Improvement

## Findings

This section describes the findings with respect to four aspects of the PExpI intervention implementation evaluation:

### Acceptability

The analysis of employee feedback around the content and experience with different training sessions indicated user-satisfaction. Specific posts were designed regarding trainings to facilitate eliciting feedback from the employees as comments. For instance, videos of leadership training workshops with highlights were posted on the Facebook group.

Participants from different cadres expressed that their experience of the training was different from the previous motivational trainings they had attended. The feedback indicated that the training was successful in engaging them and a willingness to think differently.

The “Batti factor” had arranged an extraordinary legendary leadership camp. Even though several dozen motivational training programs were arranged in a variety of fields but the one conducted by [the Consultant] has been the best in transforming us and bringing us to Game A. [Nursing staff, comment, October 18^th^, 2017].

I haven’t attended such a powerful and highly energetic leadership training program throughout my whole professional career. The impact induced by this program will not only help us in serving our valued patients in an enhanced manner but also in improving the quality of our own lives. [Senior Administrator, feedback, October 19^th^, 2017].

Some participants felt the training session was apt and it helped them connect with the humanistic side of their profession. Some shared that the session (which included a movie about compassionate patient care) helped them see things from the patient perspective and would now identify the patient with their name rather than bed number.

…To be a human in front of your patient, because that is what they expect of you. [Senior paediatric physician, comment on movie, November 6^th^, 2017].

Identify my patient with their name, not by their bed number. [Physician, comment on movie, November 6^th^, 2017].

### Adoption

With reference to the trainings, the participants also shared that they felt motivated to achieve their goals and could now make them tangible, and achievable (expressed intent to use).

A lot of times we know what we want. Some of us are also aware of the purpose. It is only when we align the two with actions that success happens. Focus is the name of the game and frequently returning to your RPM will help as will a mentor who can provide constructive feedback and encouragement. [Faculty member, comment, October 24, 2017].

The feedback on the Facebook group after the training also suggested a strong intent expressed by the employees to apply the learning and find innovative strategies to improve patient care with several participants regularly sharing progress of their QI projects on the Facebook page.

“Dr XX is telling us about his RPM [QI project] focusing on residents and fellows involvement in research” (Faculty physician, post, January 22^nd^, 2018).

“Rocking Operation Room to Cardiac CU/Pediatric ICU handover RPM by Dr. XX.” (Faculty physician, post, February 13^th^, 2018).

Also, during early implementation, a total of 178 application for different QI projects were received from the employees indicating their willingness to improve the service. The applications were reviewed by the working group for priority of the question addressed for patient experience, feasibility in terms of resources available and individual ability to lead the project. After being shortlisted, 45 were presented by respective employees in 12 different individual-focused leadership sessions. The sessions were attended by 374 staff members. As a next step, 40 applications were further shortlisted based on the same criteria. 33 of these 40 had individual follow-up meetings with the leadership coach. However, only 18 projects of these were part of final execution, which covered themes of compassion, communication, coordination, and competence (as identified from the qualitative analysis of patient feedback forms).

### Fidelity

Degree and quality of execution across the intervention strategy for each of the four enablers was used to determine the fidelity of the PExpI intervention.

#### Purpose and vision

The new vision was communicated through workshops and on a shared platform-the Facebook group by the working group members. The attendance of employees for various leadership training workshops conducted between October 2017 to March 2018 is outlined in Table 4. A total of 10 sessions were conducted for leadership trainings. The leadership training workshops were designed to cover all staff of the SL and about 90% were covered (*N* = 455). Performance on social media in the first 10 weeks indicated 97 posts and 1563 comments discussing the purpose and vision of the initiative with about 20–22% of the total posts by the working group members.

**Table 4:** Degree of execution of leadership sessions indexed by attendance per cadre

	**Session**	**Total sessions**	**Timeline**	**Faculty**	**Trainee Physician**	**Nurse**	**Admin**	**Other**	**Total**
1	Leadership Training Workshop (2 days-½ day)	10	Oct-Dec 17	42 (9.2)	59 (13.0)	287 (63.1)	54 (11.9)	13 (2.8)	455
2	Business Training Workshop (2 days)	2	Nov 17	20 (42.6)	6 (12.8)	16 (34.0)	5 (10.6)	-	47
3	Individual-focused leadership Meetings (1 h)	12	Nov 17-Jan 18	99 (26.5)	73 (19.5)	134 (35.8)	68 (18.2)	-	374
4	Group project execution meetings (1 h)	8	Jan-Mar 18	83 (29.2)	94 (33.1)	69 (24.3)	38 (13.4)	-	284
5	Individual project execution meetings	63 (with 33 employees)	Feb-Mar 18	38 (60.3)	5 (7.9)	18 (28.6)	-	2 (3.2)	63

Note: Other includes therapists, research staff and housekeeping staff

Data is reported as N (%)

#### Engaging managers

The first set of trainings for middle managers was attended by 47 people with 50% of the physicians [Table 4]. Several practical ideas for execution were discussed online and as part of the workshops on the Facebook group but could not be systematically recorded. For the second set of trainings for the managers, a complete mentorship package with a standard protocol was created for nurses. A total of 680 man-hours were spent indicating the effort put into developing this package. Quality of the final package can be judged from the level of details to ensure implementation which included the supervision checklists operationalizing compassion for both nurses and their supervisors for objective ratings [33]. Selected staff (*N* = 33) with 52% nurses in supervisory roles completed the trainings. An outcome of quality of execution of these trainings was the ideas generated leading to subsequent QI projects, but data about the QIs that emerged could not be systematically maintained. Notable QI projects from these specific trainings for engaging managers were improving the experience of undergraduate students rotating in the service line [39] and designing wellness programme for trainee physicians [40]. However, the mentorship package could not be rolled out for the frontline nursing staff during the course of the initiative.

#### Employee voice

The communication strategy utilizing social media (Facebook group) was found to be effective to engage the employees (90% active members). Several ideas were generated through the online discussions which culminated into QI projects. A detailed evaluation has been published earlier [33].

With regard to QI projects, an effort was made to ensure quality of project designs through regular dissemination about the use of ToC and conducting a one-day workshop to support feasible yet effective designs. Some of the QI projects managed to present a ToC and internal mechanisms to track progress as an indication of the quality of execution. Eight group meetings for execution were also conducted, attended by different cadres of employees (*N* = 284) where progress of the projects was discussed, and feedback was provided. The quality of execution of the QI projects was determined by the coach’s rating of them based on their progress shared in the individual meetings. According to the subjective progress notes by the leadership coach in the individual meetings for these projects (*N* = 63), about 17% (*N* = 11) showed ‘much progress’, 37% (*N* = 23) showed some progress while the rest had no progress 41% (*N* = 26) with respect to moving toward implementation. Optimal quality defined as a QI project which had clear ToC, process metrics and outcome metrics defined and were on track (all criteria need to be met to be called optimal). None of the projects was rated as showing optimal progress.

As indicators of degree and quality of execution, the following QI projects were implemented and sustained for at least one year: play-based psychosocial stimulation program [41], streamlining admission process [42], hands-off between intensive care unit and surgery operation theatres [43], improving medical students’ experience rotating in the service line [39], restructuring resident research program and “one physician model” (general pediatric service to be done by one admitting faculty at a time per week for better continuity of patient care). The sustained QI projects that tracked outcomes disseminated the results indicating benefits and quality of outcomes. The authors believe sustained execution is more attributable to individual factors (like perseverance, the ability to execute, building relationships with the team members) as organizational emphasis on execution of QI projects was reduced in the post intervention period.

#### Integrity

In terms of quality of execution, data indicated that it took an average of 4.7 days for the coordinator to share patients’ feedback with the manager and an average of 3 more days to reach the employees (excluding faculty physicians). A total of 36% of the forms were meeting the SOP of appreciation being shared within 1 day. 72 appreciations were received which had named the faculty physicians. 100% of these appreciations were emailed to respective physicians by the SLC with a personalized message in less than a day with 56 physicians responding to the email.

On the Facebook group, a total of 82 posts were uploaded for ‘person of the week’ appreciating employees for their compassionate practices towards their fellow colleagues and/or patients. Criteria for nomination was created and shared on the Facebook group by the patient experience directorship team which included: follows rules and policies (punctuality and regular attendance in meeting), demonstrated commitment, contributes to meetings/discussions, takes care of fellow colleagues/patients, is courteous, and appreciative of others’ success. This also gave an opportunity to acknowledge those not directly involved in patient care (e.g., security guards and housekeepers) but who were important in supporting those providing direct patient care. These nominations were given by peers and colleagues to the patient experience directorship team who made the final decision based on their observations and informal feedback from the supervisors and other colleagues. These appreciation posts garnered most engagement as indexed by number of likes and comments [34].

### Facilitators and barriers of implementing the PExpI intervention

The following emerged as the main facilitators during the analysis: participation of leadership, effort towards creating value around the initiative, building strategic partnerships, an employee championing the intervention, transparency of communication, real time engagement platform, and attention given by leadership to resolve challenges [Table 5].

**Table 5:** Implementation facilitators and barriers

**Facilitators**
Leadership involvement I am reading the Batti Factor posts everyday and I am very impressed by the commitment that I sense. It’s strong, it’s promising and it’s convincing. You should not doubt that education and patient care are mutually exclusive. It goes hand in hand. Just be smart, coordinate and act from your purpose. Talk, question, debate and then stick to how to do it. Organise, monitor and evaluate. If the passion is there: it will have a HUGE impact! Thank you! [CEO, post, 2nd November, 2017] I have visited for half an hour for today's RPM meeting. I was impressed. Faculty now working on standardisation of care. Why? Following best practices, evaluating them, providing best care across faculty and residents. Documenting and justifying additional tests or treatments. Why is this good? Because we can have a deeper insight into what we are doing and why. We can also provide more access by saving costs to the patients/families. We can publish our best practices. We can be soooo good! [CEO, post, 21 Nov, 2017] Who can provide more compassionate, empathetic care than a nurse? Who can be more of an advocate for their patients than their nurse? What can we do from tomorrow to make sure that none of our rounds happen without our nurses? None of our plans are made without our nurses’ input? Can people describe this picture in just one word? I describe it as Yohsin. [SLC, post, December 2nd, 2017] Priority RPMs. XYZ’s survey has shown 6 main points we need to address with our RPMS: Compassion, Competence, Communication, Quick Response, Coordination and Cleanliness. We will tailor all RPMs to ensure that they hit one of these 6 priorities. Let's begin the conversation by talking about COMPASSION. [Senior faculty lead, Post, December 15th, 2017] I am extremely proud that the Batti Factor keeps moving all of us. This is the most remarkable initiative in Pakistan healthcare ever. But allow me to give some feedback based on data that we receive [about infection rates]. May I request all of you to check via a RPM what we can do to slash these numbers? I know you have lots of issues to manage but this one needs to be addressed with urgent priority. Just my humble request to you guys who can turn this around. I am sure! [CEO, post, July 4th, 2018] Appreciating the staff challenge…I appreciated the admission office staff for doing such a tough job…I appreciated our tech and told her that she is a person with Yohsin (grace, generosity and excellence)..I challenge … to write about 2 people they will appreciate tomorrow and what did they say? I also challenge them to challenge their other friends and colleagues. Let’s see how big this movement can become? [SLC, post, October 25th, 2017]
Real-time feedback and encouragement Thanks for listening, Hans. I also lead the UG Paediatrics program and feel the Batti is missing in education as well. We will ignite it. [Physician, comment on post by CEO, 21 Nov, 2017] Why is it absolutely critical to get clear about your PURPOSE, your WHY, your driving force; resourcefulness is the ultimate resource because it allows you to transcend any limitation! When you execute your RPM's remember that any limitation is only in our own mind – period. [Consultant, post, December 16th, 2017] This transformation has always been about people solving their own problems rather than expecting someone else to come and do it. It's been a fantastic effort by all the teams and we are seeing the results on the ground. [Senior faculty manager, comment, March 28th, 2018]
Value creation Amazing Skype call with Patch Adams. He is so excited about coming down to see us on April 28th and 29th. His message to the Children's Hospital Staff, "You are the kind of team who because they take so much joy in caring, go home not burnt out, but on fire!" [Senior physician, post, March 14th, 2018] Karen Armstrong to speak about work at Children's Hospital. All the more reason now to work even harder and to live upto the expectations we have created. [Faculty, post, October 3rd, 2018] This is indeed a proud moment for all of us in the Children's Hospital Service Line. Thanks to the team of play therapist and physical therapist for their active participation and the entire team of Nursing for their marvelous work…Thanks to our faculty, fellows, residents and administration for their great support. The kind of compassionate work you all are performing was very well acknowledged by Dr. Karen Armstrong and the AKUH leadership. Superb Team and it was a well-deserved recognition. [Senior administrator, post, September 25th, 2018]
Assigned personnel Today I want to appreciate a true leader among us. Someone who has selflessly owned this transformation, has led it from the front and even now is relentlessly putting her heart and soul in keeping everything about this transformation (TOCs, RPMs, Batti page, appreciations etc.) alive and thriving. Thank you XYZ for being that “crazy one” who will change the world. [SLC, post, July 11th, 2018] You are turning out to be our biggest advocate. Your being emotionally invested truly inspires us. Employee satisfaction is deeply rooted in the workplace environment. For me real satisfaction does not come when I have a high pay scale or enjoy a title. What matters is how I am treated; how my work is appreciated; Am I treated like trash or given deserved respect, Am I always dictated or am I listened to sometime? Does a person always want to feel proud of what he does? And how would we know that we have done something that we should feel proud of? That's through appreciation and recognition by the leaders. [UR, response to a facebook post, August 19th, 2018] She is diligent in what she does and I've never met someone who is this much passionate about compassion. And she is affecting the lives of many people with her compassionate drive whether directly or indirectly. [Research associate, comment, September 13th, 2018]
**Barriers**
The one thing that I observed during the presentations and the discussions afterwards is that it seems difficult to describe the goals we want to achieve. We are good at describing the "ideal", like: "enjoyable body language and or behaviour" but the challenge is to define this in a more "smart" way. Because: what exactly is that behaviour that we want to show to our patients and their families? What is "enjoyable"? You and I can have a different understanding about this! The group will work on some short (40 s) smartphone made videos to SHOW and DEMONSTRATE what they understand is enjoyable behaviour. The second presentation I was able to attend was about clinics performance. That is a theme that we really need to pick up. Overcrowding and subsequently long waiting times are an issue as we all know (and not only in the Children's Hospital!!). This is a complex issue and needs to be analysed down to all possible root causes. Then a good approach will succeed and bring transitional improvement. [CEO, post, December 19th, 2017] I truly agree with this…..we need to select those variables which are measurable and more specific. For eg… anxiety, depression, satisfaction, enjoyment, aggression etc..there are many definitions for each of the variables and different tools are there to measure them…What we need to look for is what is applicable in our setting. [Physician, response to the above facebook post, December 19th, 2017]

*Note: CEO* Chief Executive Officer, *SLC* Service Line Chief, *RPM* Rapid Planning Method

#### Leadership involvement

An important facilitator of the initiative was participation of hospital CEO and SLC in the initiative-related workshops, sessions, and meetings. Moreover, a continued visibility was demonstrated on the Facebook group through their posts and comments ensuring engagement and motivation of the employees. The posts entailed encouragement of the participants after attending their sessions, providing progress updates, asking thought-provoking questions, and providing constructive feedback when needed.

I just want to share the number of patients’ and parents’ appreciations have drastically increased. This is most encouraging. [CEO, post, April 1st, 2018].

“The Children's Hospital is gearing up for the most unique event in its history…….Clowning with Patch Adams!!” [Senior faculty manager, Post, April 8th, 2018].

#### Real-time feedback and encouragement

Leveraging the social media technology allowed for employees in different units to connect, provide feedback, and offer views on issues/challenges raised, while also encouraging them.

This transformation has always been about people solving their own problems rather than expecting someone else to come and do it. It's been a fantastic effort by all the teams, and we are seeing the results on the ground. [Senior faculty manager, comment, March 28th, 2018].

And let's acknowledge all the thoughtful contributors who have given their heart and soul to keeping this page alive and kicking. It's a wonderful thing we have created, and we will nurture it so that it continues to thrive…. Just like we care for our patients! [Senior physician, post, April 6th, 2018].

#### Alignment between leadership’s say-do

Another facilitator for implementation was keen interest from leadership in listening to employee challenges and acting promptly to resolve them. An example was the nursing pain point of excessive documentation which was noticed by the CEO on the Facebook conversations and immediate meetings were called with hospital business process re-engineering team. The team helped restructure and revise documentation forms in the subsequent few months.

“A very fruitful conversation with the CEO regarding reducing the nursing documentation. In this presentation, eight areas of double documentation were discussed and strategized for plan of actions, exemption on the basis of JCI requirement were discussed. Shared the immediate and long-term plans according to the double documentation.” (Communication Consultant, Post, February 8th, 2018).

#### Immediate recognition and visibility of junior staff

The communication platform perhaps provided a much-needed opportunity for employees to be appreciated and duly recognized in a prompt manner in the presence of hospital leadership.

“Being a front-line staff, we receptionists are reflective of AKUH vision. Sitting at front desks we are the primary point of contact. All unit receptionists' works are alike but vary slightly when it comes to critical areas.” [Unit Receptionist, post, September 14th, 2018].

“Thank you, XX, for this wonderful post. This is indeed guiding principles for all your colleague URs. If you don't mind, I would like to pass this on to people where your thoughts can be shared more widely. Thank you for your commitment and sincerity”. [CMO, comment on the above post, September 14th, 2018].

“XX (UR) can you see the power of communication. One changes the course of cadres, institutions and the world through sincerity, honesty, communication from the heart and hard work.” [SLC, comment on the above post, September 14th, 2018].

#### Value creation

The team consciously made efforts to enhance the value of the initiative for the employees by showcasing the work internally and also inviting renowned celebrities to witness the activities in the wards. A 2-day visit for Dr. Patch Adams was arranged in April 2018 which included different activities for staff and patients. Thereafter was Dr. Karen Armstrong, who is a religious thinker, author, and the executive of Charter for Compassion, a global peace initiative. During her visit at the Aga Khan Centre in London in September 2018, she delivered the ‘Annual Pluralism Lecture’ in which she also spoke about her initiative: “Twelve steps to a compassionate life”, being implemented for a program in the pediatric SL to help the nurses and doctors develop a conducive relationship. She quoted the initiative as ‘*compassion coming from the Muslim world*’ [44]. In 2019, national celebrities like Shehzad Roy and Mehwish Hayat also made individual visits to the Children’s Hospital [45, 46]. Employees with outstanding performances were given a chance to meet these celebrities, which not only boosted their morale, but also made them feel valued.

#### Assigned personnel

Sustainable implementation of the strategies was facilitated by personnel who designed and led the interventions and were responsible for the outcomes. The office of Director Patient Experience of Care was created, and the behavioral implementation scientist was appointed as the director patient experience of care. Other working member included an organizational psychologist (first not only in the service line but at AKUH too).

#### Strategic partnerships

The team was cognizant of the fact that a sustainable impact is achieved through strategic partnerships and therefore stakeholders with a shared vision were identified. One of them was Charter for Compassion (CfC) Pakistan who co-designed the employee mentorship package and organized celebrity visits through their contacts. Another was with a psychology department of a local university as implementers of play-based therapy which was one of the QI projects [41].

#### Timely dissemination

Regular dissemination to ‘spread the word’ both internally and externally during the course of the initiative (2 manuscripts, 11 conference abstracts, 3 online case studies, 2 inter-department talks, an online blog for an international patient experience institute, thesis of an international student) was another strategy the team felt, facilitated implementation. The dissemination activities acted as means of receiving peer feedback allowing for refinement of the idea and establishing credibility of the work. Thus, motivating the employees.

### Barriers

The barriers identified during individual meetings for project execution included variation in engagement of the employees leading them, additional responsibility (more than what employees would have expected), logistical challenges, and lack of time. Additional challenges realized were the QIs not being aligned with job description of the person leading it, lack of project execution skills, and lack of institutionalization of incentives, especially for the senior managers (Table 5).

Additional human resources required were a leadership coach for 6 months and a communication consultant for 3 months. Post intervention period, a team of patient experience was created including a director of patient experience (who had expertise in implementation of human experience projects), an organizational psychologist to support strategy design for employee engagement, and 2 RAs for data collection, management and analysis. Resources in terms of costs were incurred for training workshops and execution of the QIs but were not systematically maintained.

## Discussion

The study aimed to describe the evaluation of implementation of a leadership strengthening intervention that had shown efficacy in improving family experience outcomes in the paediatric service line of a private pediatric center in Pakistan [25]. The intervention employed a collective approach for building leadership skills for all, including those not in leadership roles employing a mix of sessions and online communication. The findings suggested acceptability and adoption by the participants of the PExpI interventions. The facilitators included continual visibility of leadership, involvement, encouragement, and feedback. Timely and objective recognition of achievements also served to engage employees. A similar practice-model from the National Health Services, Scotland utilizing coaching, mentoring along with short classes and service improvement projects as a context-sensitive approach to leadership development, was found to be successful [47]. We believe the pragmatic approach of the study allowing managers and employees to practice leadership skills while on job was innovative. The QI projects were also aimed to be aligned with their job responsibilities as much possible to facilitate uptake.

We believe embedding the training programme in a shared purpose of not just improving the patient, but an overall human experience played a significant role in engaging employees with the strategy. Shared purpose is also one of the most important factors revealed by literature review along with skilled facilitation and social psychological safety, activity integration into organizational procedures, organizational support and supportive external monitoring to engage employees [48]. An international non-government organization in Nepal used a similar purpose-driven approach for successfully shaping their values and developing a culture of support [49].

An innovation of the study was the use of technology for real-time connection with employees and fostering organizational leadership development. Using social media may have encouraged internal stakeholders (employees) to emotionally engage with the vision and feel a sense of community within the organization, thus helping to evolve an organizational development strategy. Feeling being heard and supported through constructive feedback helped shape trust and cooperation. A similar feeling of the social support being the most important was expressed by a group of managers who were part of action learning in two different studies from South Africa [50, 51].

Open communication, including regular appreciation and recognition, can lead to reduced fear of supervisors and presence of social support necessary to reduce stressors and strains as indicated on the qualitative data reported. This has also been recognized by Schwarzer and Knoll (2007) in their study of the functional role of social support within the stress and coping process [52]. Another study revealed that facilitating upward communication and remaining approachable is a trait found in award-winning CEOs of healthcare systems [53]. Encouraging collaborative working and honest dialogue is a key step in preparing leadership to respond to employees’ concerns and should be continued as a strategy. Another advantage of the online communication might have been transparency as the conversations/discussions were visible to the members. Not all may have participated but could pick up the leadership vision of patient experience and human experience at large through the online conversation. Results from study in the US showed that employees’ use of internal social media was associated with an enhanced level of perceived transparency of the organization which in turn influenced their engagement [54].

A large number of QI applications received (*N* = 178) for volunteering QI projects was an indication that the intervention enabled engagement of employees. However, not many implemented projects were sustained for at least 12 months. There could be several explanations. Behaviours in any organization are maintained by incentives. For the current initiative, the incentives were not aligned with existing performance structures within the academic institution. Supporting the physicians to find value to execute projects for additional academic outputs would probably have resulted in sustained implementation. There was also a lack of focused intervention for the physicians which may have hampered their sustained engagement. A recent scoping review of interventions to strengthen leadership competencies similarly concluded that institutionalization should be an early consideration [55]. It was also realized that some projects were initiated just to be part of the initiative rather than genuine recognition of the patients’ true pain. Another major challenge realized was change in the individual management style of managers in a short-time frame. This aspect needed a long-term strategy.

It needs to be realized by healthcare settings that emotional engagement does not occur on its own, rather, it needs to be driven in employees which can be achieved through innovative leadership development strategies. A relief can be that cultural change does not require major financial investments but needs driving and supporting passion in employees, which can mean taking tough decisions in the face of resistance to change. Though faced with serious resistance during the change process, the present initiative finally culminated as a top priority of the hospital leadership to be rolled out in other service lines. For this purpose, a Patient Experience Committee had also been formed as part of a strategic plan to design the institution-wide strategy for intervention and measurement being implemented across all service lines at the institution.

There were several lessons learned during the process. Firstly, it must be realized that cultural transformation is a cumbersome process owing to the characteristic resistance and apprehensions for changing set ways. This challenge has also been highlighted by experts in developed countries where engaging physicians can be difficult due to apprehension of losing autonomy [56, 57]. A pragmatic model of change is needed at the onset informed by a robust implementation framework (ToC in the current study) to ensure clarity of the process. Once a clear ToC is developed, efforts must be directed at mapping clear process evaluation for each intervention and creating clear protocols around it. The team acquired this learning heading forward with the initiative over the two years. Some strain was experienced in making the working group committee understand its value. Sustainability of efforts necessitates recognition of the importance of data and process evaluation. To avoid this, clear guidelines must be laid out and the team must be provided with continuing education opportunities to better understand process evaluation. Evidence from leadership development programmes in LMIC health systems has also clearly demonstrated the role of implementation research in supporting such initiatives [17].

Secondly, to multiply influence and expand opportunities for continual learning, research, and process improvement, rapid collaborations with different bodies is useful e.g., partnership with a CfC Pakistan with a similar vision of compassionate care led to using their strengths for delivery of the intervention strategy and also value-creation to engage employees. On the other hand, partnering with a psychology department provided a much-needed workforce innovating to meet both supply and demand needs. The work was shared with international Patient Experience bodies like the Beryl Institute and helped the first author secure membership of their Global Advocacy Council. This also helped to capitalize on the concept of dissemination to create value. This concept incorporated the use of other platforms than just academic journals to disseminate the transformative work. To date, the work has been disseminated at leading international patient experience conferences and case studies of the work that emerged from the initiative have been published. People like to celebrate quick wins and these help to sustain engagement [58].

Thirdly, translating concepts like ‘compassion’ into observable and measurable behaviors is critical to reducing ambiguity. The team ventured into an innovative approach to practically incorporate compassion in nursing care and an effort to quantify it via nursing mentorship checklists. This was achieved in collaboration with CfC Pakistan who are the key local players in striving to restore compassionate action in the society. The final checklists may not be perfect but will evolve as they are implemented [33].

Fourthly, in the case of hiring an external coach to guide the transformation process, it is compelling to oversee an equilibrium of external and internal input. During the study, the team learned this valuable lesson in the wake of contrasting approaches at the onset. It is important to be mindful of internal expertise owing to extended awareness of internal mechanisms and structures. The need of empowering internal actors to function as transformative leaders in the change initiatives has also been highlighted by other studies [17, 22]. Also connected to this point is that the working group leading the change process must be carefully chosen with the right person for the right job emerging from the conceptual framework. An effective working group should ideally be composed of people in leadership and decision-making positions and those with expertise in implementation science.

Lastly, effort must be made to institutionalize the interventions by ensuring job alignment at the outset and designing incentivization. One way of doing that can be to create value for QI project by adding to the routine performance reviews and appraisals﻿ [59–61].

The qualitative data analyzed is based on about 9000 posts and over 60,000 comments by the employees on the social media group (Facebook). We believe this is an innovative approach to collecting ongoing and real-time feedback. Free expression of thoughts initiated by the participants themselves and a fluid discussion may be a more appropriate indication of the current thought process as opposed to conducting interviews in a formal setting with a retrospective recollection of feedback [62]. A notable limitation however is that since we utilized employee feedback from the social media platform, the data may have been biased toward employees who were willing to post or who may have posted only positive comments. We have tried to complement the data with quantitative indicators for a balanced approach. Another limitation was the fact that we could not present data by individual employees or by different cadres due to unavailability of data owing to changes in data access rules by Facebook. The Facebook group was created as per advice of the leadership coach to communicate the updates of the initiative with the aim to influence the employees, but it organically evolved as place where employees were comfortable sharing their challenges, achievements and project progress. The platform may raise ethical concerns and a secure platform for employee recognition was underway in the institution as an endavour led by the human resources department.

Another limitation was that we did not have any objective data of the quality of the QI projects during implementation and were based on the coach’s understanding. The study being conducted in a private, elite tertiary care urban hospital can be considered a limitation as the usual concern from academics is a lack of generalizability. However, we argue for it from the lens of innovation at scale [63, 64]. In Pakistan, private providers have a vital role to play if we were to transform healthcare services. Secondly, scaling a social innovation requires value-creation for the masses which starts from elite urban centers who are sometimes seen as the national role models and can also have a powerful influence over national policymaking. Thirdly, these centers have stronger governance and accountability structure compared to the public systems. It is a low-hanging opportunity for innovators.

## Conclusion

The authors conclude that implementation of a leadership strengthening intervention was acceptable with significant number of employees indicating willingness to adopt and lead QI projects. The leadership sessions were attended by 75% of employees receiving an orientation of the programme and the social media strategy managed to engage the employees with 90% active members. However, sustained execution was limited to six projects owing to different challenges identified e.g., the shorter duration of the initiative, incentives not being institutionalized, and QI project roles not aligned with job description. The implications include inclusion of a social media-based communication to engage employees for patient experience initiatives in healthcare settings with hierarchical leadership practices.

## Supplementary Information

**Additional file 1:** **TableS1.** Thematic analyses of patient feedback forms pre-intervention phase. **Table S2.** Intervention procedures for each enablerof employee engagement.

**Additional file 2.** 

**Additional file 3.** 

## Data Availability

The datasets generated and/or analysed during the current study are not publicly available due given they pertain to employee information but are available from the corresponding author on reasonable request.
