# Supplementary material for: Implementation evaluation of a leadership development intervention for improved family experience in a private paediatric care hospital, Pakistan
Source: BMC Health Serv Res. 2022 Jul 23;22:944. doi: 10.1186/s12913-022-08342-2 (PMC9308933; doi:10.1186/s12913-022-08342-2)
Supplement: Supplementary file 1 — Additional file 1: TableS1. Thematic analyses of patient feedback forms pre-intervention phase. Table S2. Intervention procedures for each enablerof employee engagement. [file 12913_2022_8342_MOESM1_ESM.docx]

**Intervention Development and Design**

**The LMIC healthcare context**

Changes in leadership practices to promote participatory approach encouraging staff motivation must be a focus in LMICs and special attention is needed on cultivating values like compassion and empathy on an organizational level to promote patient-centered care. This understanding must compel the existing healthcare system to take a step back from the typical authoritarian styles of governance and open itself to new learning and knowledge encircling effective leadership models. Accepting human vulnerability, encompassing the leaders should be the foundation of patient experience initiatives in healthcare settings. When governance and accountability is weak, leaders are equally vulnerable to drive the culture transformation and action learning can be an effective intervention to support the process. Similarly, when employees feel vulnerable in such settings, connecting with their own vulnerabilities and strengthening the system for them, helps them identify with the patient’s vulnerabilities. Intervention approaches need to focus on creating safe spaces to share their vulnerabilities. Hence, mentorship for both emotional strength and implementation skills is needed to fight the unstable and changing conditions in LMICs where physicians are not trained for leadership, healthcare systems have weak governance, accountability, and what exists is one-off training programs that tend to focus on management of particular disease programmes, rather than on skills to engage workforce.

**Approach to intervention design**

The pluralist model of healthcare views the notion of leadership as operating *between* people than an ability *within* people and characterized by the dynamic connections between individuals and their shared values [1]. When social relations are built through engagement in conversations it enables cooperation and subsequently serve to engage employees for action [2]. Fostering such an approach would require *an action learning* method whereby small groups of employees meet over time to tackle real problems or issues. Enhancing patient experience would mean the employees and leadership co-constructing shared values around leadership from the lens of human vulnerability to engage workforce for the common goal [3,4]. The action learning approach entails on-going reflection and iterative learning process. Described as a flexible and adaptable, the approach is recommended for the design and implementation of such training programmes with customization for a specific context [5]. An evaluation of a programme based on action learning in two health units in Mauritania revealed found that continuous capacity-building and empowerment through participatory approaches to facilitate change. A sustained engagement of dialogue with proactive leaders with a pragmatic approach were recommended to facilitate further change process [5].

**Conceptualization**

A need for improved patient and family experience outcomes was identified by a behavioural implementation scientist (with prior experience of implementing large scale studies for improved human interactions) and the service line chief (with significant experience in implementing value-based care interventions for health outcomes) in the paediatric service line at a private tertiary care center in Pakistan. Resistance was foreseen as part of the change process to transform from physician-centric center to a patient-centric one. Mere introduction of new policies without an attempt to incorporate the values into the culture would have meant failure. The intervention also needed to be *frugal* for quick buy-in, feasibility and long-term sustainability [6]*.* The need to intervene in resource-constrained, physician-centric and hierarchical context provided an opportunity to be creative. The team realized that the change process would entail a guided coalition and communicating a new vision of patient and family experience but would not be effective until employee experience was also taken care of. This required skills of effective leadership to manage a new way of working and for all.

**Formative work**

A total of 249 parental feedback forms (230 parent appreciation and 19 complaint forms) received between November 2016 to May 2017 were analyzed by the first author (MR) and a research associate using an inductive approach. Six themes emerged from the analysis suggesting patients’ perception of quality of care [Table 1]. Next, a quantitative survey with families of children admitted in the paediatric acute care ward was conducted to elicit feedback about their experience. The findings from 221 families revealed responsiveness (37.9%), communication (29%), and seating space for the attendant (21%) to be the top 3 priorities of these families. The factors causing most stress included: child’s pain (79.1%), treatment costs (43.2%), and inadequate explanation about labs and illness (40.9%).

Table S1: Thematic analyses of patient feedback forms pre-intervention phase

| **S #** | **Theme** | **Quotes** |
| --- | --- | --- |
| 1 | Compassion | *All the staff are highly professional and cooperative. [APP-17-34]*  *The attitude of nursing staff was extremely casual. The doctors on duty were not professional enough. [QA-17-178]* |
| 2 | Competence | *I am completely satisfied with the treatment and care. [APP-17-133]*  *We really think that sepsis could have been diagnosed earlier. [QA-16-170]* |
| 3 | Communication | *I found him a courteous person with an excellent explanation of what he is doing. [APP-17-36]*  *The physicians reported different views on my patient leaving us confused. [QA-17-05]* |
| 4 | Responsiveness | *Doctors and nurses have always paid immediate attention to our needs. [APP-17-05]*  *The family had to call the staff multiple times to notice the patient… [QA-17-316]* |
| 5 | Coordination | *Internal processes and communication within departments is effective*. [APP-17-186]  *I was informed that the lab forgot to do the test G6pd. It is about my child. She is not a toy!! [QA-17-02]* |
| 6 | Cleanliness | *Atmosphere is good and clean. [APP-17-200]*  *Your ward was not properly clean. [QA-16-354]* |

Note: the key is APP/QA=appreciation or complaints- 17 (year) followed by assigned ID

**Design of intervention strategies**

Implementation feasibility and acceptability was a key consideration of the intervention design to mitigate risk of resistance to a behaviour change intervention. Also, an approach that helped to reduce the complexity of implementation by breaking the intervention into intermediate measurable steps, rendering better evaluation possibilities of the behaviour change intervention was preferred. Hence, we employed the theory of change (ToC) , “*an approach which describes how a programme brings about specific long-term outcomes through a logical sequence of intermediate outcomes*” [7]. Using this approach, we set to explain not just how but why the intervention enablers would work in the organizational context.

**Interventional Procedures**

The intervention had 4 enablers described below and summarize in Table 2.

Purpose and vision:

The process aimed to help individuals realize- in a confrontational style, what motivated them, what were the barriers to motivation, and how could those be overcome asserting that the decision one takes and not the environment is the key to transformation [8]. A review of improvement attributes in healthcare also found that engaging employees through helping them find a meaning that aligns with their professional loyalty was more effective than enforcing regulation. The approach overlaps with transformational style of leadership, which is characterized as autonomous, honest and empowering [9]. This approach was deemed fit for the initiative to manage change and also address resistance.

Engaging managers:

The training workshops to engage managers included sessions to help participants empathize with patients e.g., physicians calling helpdesk and scheduling an appointment for themselves pretending to be a patient to realize the pain during the process. The workshop on compassion and mentorship focused on teaching managers in how to engage employees effectively through use of mentorship. These workshops were done in collaboration with a partner organization, Charter for Compassion (CfC), Pakistan (a local initiative inspired by the Charter for Compassion International). Standard Operating Procedures (SOP) of an on-job mentorship programme embedded in a framework of compassion were created with a focus on nursing, which formed the largest cadre of frontline service providers [10].

Employee voice:

The Facebook page posts were designed to: communicate the strategic vision and organization’s policies and values by the leadership, connect with the employees by listening to them and encouraging solutions, ensure transparency, and recognize employees providing optimal care. The strategy was designed by the working group iteratively in their weekly meetings driven by on-going engagement data. Details of the strategy have been described in another manuscript [11]. Employees were invited on the page and group sessions to propose ideas based on the themes that emanated from the parental and employee experience surveys to improve their work processes as a quality improvement (QI) project.

Moreover, QI is also seen as the core outcome of effective clinical leadership. The employees were provided assurance for support to lead the projects. To ensure execution, two levels of intervention were conducted: i) Individual-focused leadership meetings (for those who presented ideas to lead the QI). An exploration session with questions was moderated by the coach to help them engage emotionally and identify their purpose of leading a particular QI. The session was open to all employees with a live video session (Facebook page); and ii) after the selection process, project execution meetings were conducted as per need of the participants with the consultant.

Integrity:

As per the SOP, notable patient appreciations for respective employees and their unit head were recognized on email and morning huddles by head nurse and also posted on the Facebook group within 1 day by the director experience of care. On a monthly basis, compiled data were also shared.

Table S2: Intervention procedures for each enabler of employee engagement

| ***Purpose and vision*** |
| --- |
| The approach to purpose-driven trainings was built on a framework the leadership coach employed in his training with Tony Robbins (2006), a US-based business strategist. Robbins’ philosophy lays the responsibility on the individual to change one’s life and circumstance around them. Leadership training sessions were conducted based on the above approach led by the external coach. The sessions focused on explaining the notion of leadership, higher purpose, vision, taking responsibility, mastery of emotions, and a sense of purpose to create psychological readiness for change. A two-days’ workshop for the senior staff, while a half day workshop was conducted for other personnel. Additionally, the coach had weekly 2-hour meetings for on-going strategic planning and execution with the working group. |
| ***Engaging managers*** |
| Recognizing the importance of middle managers, a 2-days workshop was designed for the employees in managerial roles to facilitate a deeper dive into the patient needs and set the direction and clarity for the execution of the transformation initiative [details in Supplemental text]. Another set of training was also conducted about 7-8 months into the roll out to teach managers compassion and mentorship skills. |
| ***Employee voice*** |
| To facilitate upward communication and to listen to employees, a Facebook group was created. The page included higher leadership (e.g., Chief Executive Officer- CEO, Chief Medical Officer- CMO) and also the employees (physicians, nurses, trainees, housekeeping staff). This communication intervention were envisioned to specifically target employee experience through the feeling of being heard, access to the leadership, sharing challenges from the ground but also given the opportunity to implement them. Engaged employees were subsequently invited to lead quality improvement projects as an index of leadership skills. |
| ***Integrity*** |
| Demonstrating integrity meant valuing patient experience outcomes and high-performing employees demonstrating patient-centered care in timely and consistent manner. Thus, creation of a fair and objective recognition system was imperative. It was felt that the patient feedback gained attention only when it was a complaint and appreciations were ignored causing resentment in the employees. Therefore, a SOP was created to streamline routine patient experience data received through patient feedback forms (an institutional process). The core outcome of the SOP was to help the patient feedback reach the employee/employees appreciated by the patients in their feedback forms as soon as possible. Process metrics were designed to understand the bottlenecks.  We further developed a feedback system around it i.e., when suggestions or complains highlighted what needs to be improved and how the role needs to assign with regular monitoring through data. The data was also an opportunity to be informed of patient priorities to frame QI strategies. The SOP ensured transparency and the employees were also recognized on the Facebook group.  Moreover, the employees who were recognized were given exclusive opportunity to meet the celebrities who were specifically invited to visit during the initiative. |

**References**

1. de Zulueta PC. Developing compassionate leadership in health care: an integrative review. Journal of healthcare leadership. 2016;8:1.doi: 10.2147/JHL.S93724
2. Edmonstone J. What is wrong with NHS leadership development? British Journal of Healthcare Management 2013; 19(11): 531-538.
3. Berger U, Trehan, K. 2018. Action learning in East Africa: new encounters or impossible challenges? Action learning: Research and Practice, 15(2), pp.126-138.
4. Lehmann U, Gilson L. Action learning for health system governance: the reward and challenge of co-production. Health Policy and Planning. 2015 Oct 1;30(8):957-63. https://doi.org/10.1093/heapol/czu097
5. Accoe K, Marchal B, Gnokane Y, Abdellahi D, Bossyns P, Criel B. Action research and health system strengthening: the case of the health sector support programme in Mauritania, West Africa. Health Research Policy and Systems. 2020;18(1):1-3.
6. Jumbam DT, Durnwald L, Munabi NC, Ayala R. Implementation science: A missing link in global surgery. East and Central African Journal of Surgery. 2021;26(2):45
7. Breuer E, Lee L, De Silva M, Lund C. Using theory of change to design and evaluate public health interventions: a systematic review. Implementation Science. 2015;11(1):1-7.
8. Harnett PJ. Improvement attributes in healthcare: implications for integrated care. International Journal of Health Care Quality Assurance. 2018;31(3). DOI 10.1108/IJHCQA-07-2016-0097
9. Lumbers M. Approaches to leadership and managing change in the NHS. British Journal of Nursing. 2018;27(10):554-8.
10. Hookmani AA, Lalani N, Sultan N, Zubairi A, Hussain A, Hasan BS, Rasheed MA. Development of an on-job mentorship programme to improve nursing experience for enhanced patient experience of compassionate care. BMC Nursing. 2021;20(1):1-8.
11. Rasheed MA, Hookmani AA, Waleed S, Fatima HS, Sidiqui S, Khurram M, Hasan BS. Implementation and evaluation of a social media-based communication strategy to enhance employee engagement: experiences from a Children's Hospital, Pakistan. Frontiers in Public Health. 2021 Mar 11;9:55. <https://doi.org/10.3389/fpubh.2021.584179>
