# Supplementary material for: Implementation evaluation of a leadership development intervention for improved family experience in a private paediatric care hospital, Pakistan
Source: BMC Health Serv Res. 2022 Jul 23;22:944. doi: 10.1186/s12913-022-08342-2 (PMC9308933; doi:10.1186/s12913-022-08342-2)
Supplement: Supplementary file 3 — Additional file 3. [file 12913_2022_8342_MOESM3_ESM.docx]

1. **Introduction**

The document describes standard operating procedures for managing patient feedback by Service Line-4 after receiving from Clinical Affairs Department (CAD). The purpose for managing and systematically analyzing patient feedback will be to devise preventive strategies for improved patient experience.

1. **Team**

The table presents the team managing patient feedback with their roles.

| **Name** | **Designation** | **Responsibilities** |
| --- | --- | --- |
| AR | Business Manager | - Managing complaints regarding customer service issues and logistics. - Support strategies to prevent logistic issues. |
| BS | Service Line Chief | - Oversee the process and take final decisions. - Reviewing final responses to all complaints before shared with CAD. - Will be in loop for all correspondence with CAD and CMO office. |
| MR | Director Patient Experience | - Oversee the data analysis process-both quantitative and qualitative. - Reviewing final responses to all complaints before shared with CAD from the perspective of patient experience. - Recommend preventive strategies for issues arising and support implementation. - Will be in loop for all correspondence with CAD. - Build liaison with CAD and CMO office. |
| NL | Nursing Manager | - Responsible for complains regarding nursing. - Lead implementation of strategies to prevent issues under nursing cadre. |
| SW | Organizational Psychologist | - Work closely with MR to inform preventive strategies for a culture change informed by literature from organizational psychology. |
| ZK | Asst. Manager | - Coordinate the patient feedback component. - Manage the data (patient feedback forms). - Manage correspondence with staff and CAD. - Connects with families (who have shared the complaint) if needed - Review final responses to all complaints before shared with CAD - Analyze and prepare monthly quantitative data reports. |

1. **Process**

The process for managing appreciations, complaints and suggestions is given below.

***3.1 Appreciations***

- Email received from CAD; addressed to ZK cc to others
- ZK categorizes the form: cadre, unit and theme
- Sent to the responsible manager of staff named (and also co-chief) within 1 working day with cc to responsible team member and MR.

| **Staff Named** | **Responsible Manager** | **Responsible Team Member** |
| --- | --- | --- |
| Nurse | Unit head nurse | NL |
| Faculty | Co-chief | SLC |
| Fellow | Fellowship director  C-chief of the section | ZK |
| KResident | Programme Director and Programme Coordinator | ZK |
| Administration | Unit coordinator | AR |

- Acknowledgment received form the manager of sharing with the staff in morning huddles, expected time frame is 1 working day.

***Complaints/Suggestions***

- Email received from CAD; addressed to ZK cc to others.
- ZK categorizes: cadre, unit and theme
- ZK connects with the family via phone call based on her judgment of the ambiguity of the complaint and logs her call within 2 working days.
- ZK sends email within 1 working day after the call to the family to the respective manager of the staff/unit named with cc to the responsible Patient Feedback team member and MR. Also adds notes of her call with the family to the email.
- Respective Patient Feedback Team member will support the members for framing the response specifically for nursing and administration staff.
- Expected time frame of receiving the response: within 5 working days. If not received within 5 days, informs the Patient Complain team with the reason of delay and number of follow-ups made with relevant manager.

| **Staff Named** | **Responsible Manager** | **Responsible Team Member** |
| --- | --- | --- |
| Nurse | Unit head nurse | NL |
| Faculty | Co-chief | SLC |
| Fellow | Fellowship director  C-chief of the section | ZK |
| Resident | Programme Director and Programme Coordinator | ZK |
| Administration | Unit coordinator | AR |

- ZK/MR/BH review response within 3 working days.
- ZK shares with CAD within 1 working day.
- Time frame of response to CAD from receipt of email is within 12 working days.

1. **Data Management**

- Data will be managed using Dropbox. MR will create a folder, share invite with all team members and organize the folder into sub-folders. All the forms and subsequent reports will be uploaded in Dropbox. For quality check of the numbers of patient complain forms, ZK will cross-check with CAD. All the forms will be logged onto an excel sheet for a monthly analysis. Template has been prepared by MR.

1. **Data Analysis**

- Monthly report to be prepared by 5^th^ of every month.
- Quantitative aspects will be completed by ZK

Indicators to include:

***Appreciations***

number (%) by cadres, unit and theme

n (%) response shared within 1 working day

n (%) acknowledgments received within 1 working day

***Complaints***

n (%) by cadres, unit and theme

n (%) families called by ZK within 2 working day of receiving the complaint

n (%) complaints shared by ZK within 1 working day

n (%) responses received from respective mangers within 5 working days

n (%) responses reviewed by ZK/MR/BH within 3 working days

n (%) complaints shared with CAD within 15 working days

n (%)complaints not shared with CAD within 15 working days by cadre/unit/theme

- MR to complete qualitative analysis with recommendations and follow up status of previous recommendations
- The team decides implementation of the recommendations in a monthly meeting.

1. **Dissemination**

- Quantitative data compiled and shared with patient experience team by ZK by 5^th^ of every month.
- Qualitative analyses and preventive strategies will be shared by MR/SW by 5^th^ of every month.
- Appreciation data for each month along with staff names will be posted on the page by ZK by 8^th^ of each month.
- Appreciation data will be emailed by ZK to the respective managers so they appreciate the staff in morning huddles by 8^th^ of every month with copy to MR and respective patient feedback team member.
- A monthly patient feedback team meeting by 10^th^ of every month will be coordinated by ZK to discuss data and approval on strategies.
- Data on complaints/suggestions and action points will be shared with respective managers by 15^th^ of every month by ZK with copy to MR and relevant team member.
- Nursing data will be presented in Town Hall meetings.
- ZK to coordinate for follow-up of action points. Template for the action and follow-ups will be created once report on action points is prepared by MR.
- A quarterly report with status of actions points recommended and followed-up will be shared with CMO office.
